# Deep neck abscesses: study of 101 cases^☆^

**DOI:** 10.1016/j.bjorl.2016.04.004

**Published:** 2016-05-05

**Authors:** Thiago Pires Brito, Igor Moreira Hazboun, Fernando Laffitte Fernandes, Lucas Ricci Bento, Carlos Eduardo Monteiro Zappelini, Carlos Takahiro Chone, Agrício Nubiato Crespo

**Affiliations:** Universidade Estadual de Campinas (UNICAMP), Faculdade de Ciências Médicas, Departamento de Otorrinolaringologia, Campinas, SP, Brazil

**Keywords:** Neck abscess, Neck infection, Neck spaces, Abscesso cervical, Infecção cervical, Espaços cervicais

## Abstract

**Introduction:**

Although the incidence of Deep Cervical Abscess (DCA) has decreased mainly for the availability of antibiotics, this infection still occurs with considerable frequency and can be associated with high morbidity and mortality.

**Objective:**

This study aimed to present our clinical-surgical experience with deep neck abscesses.

**Methods:**

A retrospective study analyzed 101 patients diagnosed with deep neck abscesses caused by multiple etiologies, assisted at a medical school hospital during 6 years. One hundred one patients were included and 27 (26.7%) were younger than 18 years old (the children group), 74 patients (73.3%) were older than 18 years old (the adults group). The following clinical features were analyzed and compared: age, gender, clinical symptoms, leukocyte count, the affected cervical area, lifestyle habits, antibiotic therapy, comorbidities, etiology, bacterial culture, time of hospitalization, the need of tracheostomy and complications.

**Results:**

There was predominance in the male gender (55.5%) and young people (mean age 28.1 years). All of the 51 patients with associated disease comorbidity were adults. The most frequent etiologies were bacterial tonsillitis (31.68%) and odontogenic infections (23.7%). The most common cervical areas affected were the peritonsillar (26.7%), submandibular/mouth floor (22.7%) and parapharyngeal spaces (18.8%). In children group, the site most commonly involved was the peritonsillar space (10 patients, 37%). In adults group, the site most commonly involved was multispace (31 patients, 41.8%). *Streptococcus pyogenes* (23.3%) was the most common microorganism present. Amoxicillin associated with clavulanate (82.1%) was the more used antibiotic. The main complications of abscesses were septic shock (16.8%), pneumonia (10.8%) and mediastinitis (1.98%). Tracheostomy was necessary in 16.8% of patients. The mortality rate was 1.98%.

**Conclusion:**

The clinical features and severity of DCA varied according to different age groups, perhaps due to the location of the infection and a higher incidence of comorbidity in adults. Thus, DCA in adults is more facile to have multispace involvement and lead to complications and seems to be more serious than that in children.

## Resumo

**Introdução:**

Embora a incidência dos abscessos cervicais profundos (ACP) tenha diminuído principalmente pela disponibilidade dos antibióticos, essa infecção ainda ocorre com frequência considerável e pode ser associada a alta morbidade e mortalidade.

**Objetivo:**

Este estudo teve como objetivo apresentar nossa experiência clínico-cirúrgica com abcessos cervicais profundos.

**Método:**

Um estudo retrospectivo realizado em um hospital universitário analisou 101 pacientes, durante seis anos, diagnosticados com abscessos cervicais profundos causados por múltiplas etiologias. Foram incluídos 101 pacientes, sendo que 27 (26,7%) tinham menos de 18 anos (grupo das crianças) e 74 (73,3%) tinham mais de 18 anos (grupo dos adultos). As seguintes características clínicas foram analisadas e comparadas: idade, sexo, sintomas clínicos, área cervical acometida, hábitos de vida, antibioticoterapia, comorbidades, etiologia, cultura bacteriana,tempo de internação, necessidade de traqueotomia e complicações.

**Resultados:**

Houve predominância do sexo masculino (55,5%) e de jovens (idade média de 28,1 anos). Todos os 51 pacientes com comorbidade associada eram adultos. As etiologias mais frequentes foram tonsilite bacteriana (31,68%) e infecções odontogênicas (23,7%). As áreas cervicais acometidas mais comuns foram a peritonsilar (26,7%), submandibular/assoalho da boca (22,7%) e espaços parafaríngeos (18,8%). No grupo das crianças, o local mais comumente envolvido foi o espaço peritonsilar (10 pacientes, 37%). No grupo dos adultos, houve predomínio de acometimento de múltiplos espaços (31 pacientes, 41,8%). *Streptococcus pyogenes* foi o microrganismo presente mais comum (23,3%). A amoxicilina associada ao clavulanato foi o antibiótico mais usado (82,1%). As principais complicações dos abscessos foram choque séptico (16,8%), pneumonia (10,8%) e mediastinite (1,98%). A traqueostomia foi necessária em 16,8% dos pacientes. A taxa de mortalidade foi de 1,98%.

**Conclusão:**

As características clínicas e a gravidade do ACP variaram de acordo com diferentes faixas etárias, possivelmente devido à localização da infecção e à maior incidência de comorbidades em adultos. Assim, o ACP em adultos acomete mais facilmente múltiplos espaços, apresenta mais complicações e parece ser também mais grave do que em crianças.

## Introduction

Deep cervical abscesses (DCA) is defined by the presence of pus in the spaces and fasciae of the head and neck. DCA can be categorized into retropharyngeal, peritonsillar, masseteric, pteropalatine maxillary, parapharyngeal, submandibular, parotid and floor of mouth abscesses.^1^ Despite the improvements in diagnostic tests and the availability of modern antibiotic therapy, those infections continue to cause significant morbidity and mortality rates, especially when there is no early treatment.^2^ It occurs with considerable frequency and its severity and extent may be underestimated, making this entity diagnostic challenge to emergency physicians, pediatricians, otolaryngologists and head and neck surgeon, because clinical signs and symptoms often overlap with those of other common clinical pictures (i.e. pharyngitis, tonsillitis and torticollis), particularly in children, in whom physical examination may be more difficult than in adults. However, apparently,^3^ in adults is easier to have multispace involvement and lead to complications and appears to be more serious than that in children.^4^ In addition, use of analgesic and anti-inflammatory drugs may mask presentations. It is sometimes difficult to find the origin of DCA because the primary source of infection may precede it by weeks.^5^

DCA are potentially fatal and require an aggressive diagnostic and therapeutic management to avoid life-threatening complications, such as airway obstruction, cervical necrotizing fasciitis, jugular vein thrombosis, disseminated intravascular coagulation empyema, mediastinitis, aspiration pneumonia or thrombosis/aneurysm of the carotid artery. Usually polymicrobial, DCA occur from previous uncontrolled infections such as tonsillitis, dental infections, surgery, or trauma to the head and neck lymphadenitis after upper airways infection.^2^, ^6^ It is necessary to investigate risk factors such as infections, foreign bodies, trauma, immunosuppression and addiction to intravenous drugs. Concomitant diseases such as congenital cysts and fistulas, TB, diabetes mellitus, HIV, tumors, deficiency states and so on should also be taken into consideration.^7^ The clinical manifestations are diverse and depend on the affected cervical area. Moreover, an inappropriate use of antibiotics may change the clinical presentation of infections of this kind, making them elusive.^4^ Patients may be mildly symptomatic and present with symptoms of fever and pain, or experience more severe or life threatening symptoms such as dyspnea airway obstruction and septic shock.

This study aimed to report our clinical experience of submission of deep neck space infections by the description of 101 patients diagnosed in the last 6 years.

## Methods

We reviewed the medical records of 101 patients diagnosed with cervical infection spaces served by the Otolaryngology, Head and Neck surgery from a Brazilian Hospital of Medical School during the period from January 2007 to January 2013. All patients signed a document authorizing the use of data from their records (that is a standard hospital procedure).

In all cases, the patients underwent a surgical procedure to drain pus. The diagnosis of deep neck abscess was suspected by clinical history and confirmed by Computed Tomography (CT) or surgery. This analysis excluded patients with cervical infections who did not require surgery, such as cellulites, and superficial or limited infections. All patients were submitted to antibiotic therapy and, when possible, samples for culture and sensitivity.

We analyzed and compared the following clinical features: age, sex, clinical symptoms, leukocyte count, cervical area affected, lifestyle habits, antibiotic use, comorbidities, etiology, bacterial culture, length of stay, need for tracheostomy and complications.

All descriptive data were reported in percentages. Statistical evaluations were performed with a 2 sided *t*-test corrected for inequality of variances and degrees of freedom. Fisher exact test and *χ*^2^ test were used to compare the categorical variable. SPSS (13.0, SPSS Inc, Chicago, IL) were used to analyze the data and a *p*-value <0.05 was considered statistically significant.

## Results

### Population

**Figure 1:**
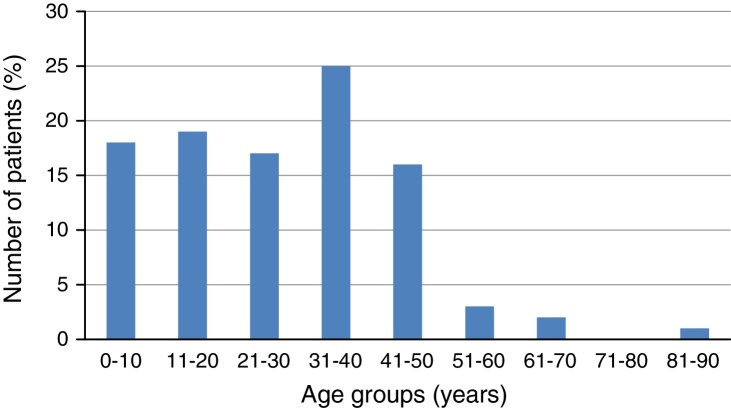
Subjects distribution according to age.

Of the 101 patients, 56 were male and 45 female, 55.5% and 44.5% respectively. Their ages ranged from 1 to 81 years with a mean age of 28.1 years. Most patients were young, the 2nd and 4th age groups. Among children, the mean age was 8.4 years, with a slight male predominance (57% of patients) (Fig. 1).

### Symptoms and time to diagnosis

The most common symptoms at diagnosis were fever (86.1%) and neck pain (81.1%). Other symptoms were odynophagia (75.2%), cervical edema (60.3%) and trismus (47.5%). Thirteen patients (12.8%) had symptoms of airway obstruction, eight males and five females with a mean age of 31.3 years. Fifteen patients (14.8%) had signs of bacteremia at the time of diagnosis, and those collected blood cultures. The mean time from symptom onset to the demand for health services was eight days (range 2–20 days).

### Lifestyle and comorbidities

Nineteen patients (18.8%) were smokers, 24 patients (23.7%) alcoholics and 10 patients (9.9%) drug users. The most prevalent comorbidities were hypertension (19 patients, 18.8%), diabetes *mellitus* (DM) (13 patients, 12.8%). Other less prevalent comorbidities were obesity (10 patients, 9.9%), hypothyroidism (4 patients, 3.9%) and hepatitis C (3 patients, 2.9%). In two patients there was an association with HIV (%). The adult patients exhibit more comorbidities than children (*p* < 0.01) (Table 1).

**Table 1:** Comparison of children and adults.

	No. of patients	No. of multispace	No. of complication	Death	No. of comorbidity
Children	27	2	8	0	0
Adults	74	31	40	2	51
*p*-Value		<0.001	0.005	>0.10	<0.001

### Etiology

**Figure 2:**
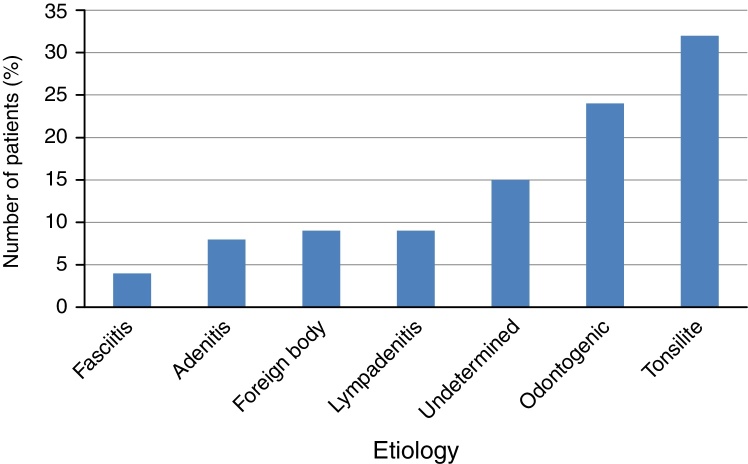
Etiology of deep cervical abscesses.

Bacterial tonsillitis was the most common cause of cervical abscess (32 patients, 31.68%), followed by odontogenic infection (24 patients, 23.7%). In 15 patients (14.8%), the cause could not be identified. Other etiologies were: post-upper airways infection, lymphadenitis and foreign body ingestion (9 patients each, totaling 17.8%), adenitis (submandibulite: 6 cases, 5.9% and mumps: 2 cases, 1.9%) and fasciitis (4 patients, 3.96%). In 58.3% of cases of etiology related to odontogenic infection there was a history of recent dental manipulation (Fig. 2).

### Spaces and cervical lymph nodes

**Figure 3:**
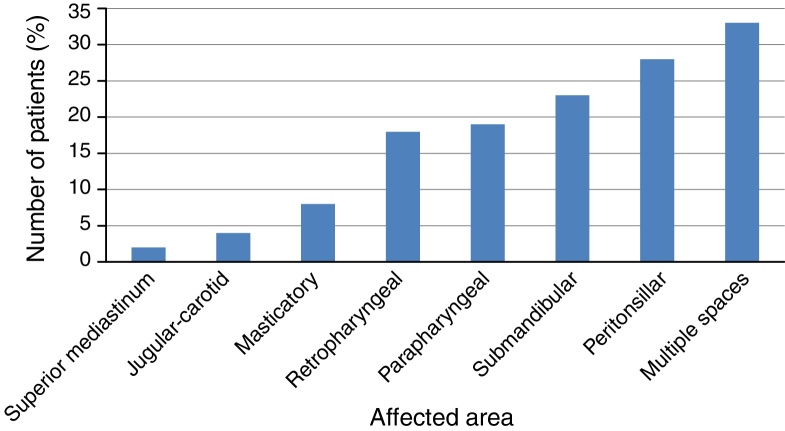
Extent of infeccion by deep cervical abscesses.

Neck CT scan was performed to diagnose and assess the extent of infection in 71.2% of patients. The remaining patients had disease extension intraoperatively detected. Peritonsillar cervical area was the most affected in 26.7% of cases (27 patients). The other areas affected in descending order were: submandibular/floor of mouth (23 patients, 22.7%), parapharyngeal (19 patients, 18.8%), retropharyngeal (18 patients, 17.8%), chewing (8 patients, 7.92%) and jugular-carotid (4 patients, 3.96%) (Fig. 3).

As Table 2 shows, the site most commonly involved in children group was the peritonsillar space (10 patients, 37%), followed by parapharyngeal space (9 patients, 33.3%), the submandibular space in 4 patients (14.8%) and the retropharyngeal space in 4 patients (14.8%). In adults group, the site most commonly involved was multispace (31 patients, 41.8%), followed by submandibular space in 19 patients (25.6%), the peritonsillar space in 17 patients (22.9%). The adult patients developed multispace infection more often than the children (*p* < 0.01) (Table 1).

**Table 2:** Distribution of the sites of deep neck abscesses.

	Children *n* (%) (*n* = 27)	Adults’ *n* (%) (*n* = 74)	Total (*n* = 101)
Multispace	2 (7.4)	31 (41.8)	33 (32.6)
Parapharyngeal space	9 (33.3)	10 (13.5)	19 (18.8)
Peritonsillar Space	10 (37)	17 (22.9)	27 (26.7)
Submandibular space	4 (14.8)	19 (25.6)	23 (22.7)
Retropharyngeal space	4 (14.8)	14 (18.9)	18 (17.8)
Masticator space	0	8 (10.8)	8 (7.92)
Jugular-carotid space	0	4 (5.4)	4 (3.96)
Upper Mediastinum	0	2 (2.7)	2 (1.9)

There was the occurrence of multiple cervical spaces in 33 patients (32.7%). When present, lymphadenopathy most often reached levels II and III. There was extension to the superior mediastinum in 2 patients (1.9%).

### Bacteriology and leukogram

All patients received antimicrobial therapy. Amoxicillin + clavulanate was the most used antibiotics as first-line treatment (82.1% of cases), followed by ceftriaxone plus metronidazole combination. The change of antibiotic depended on the culture results or clinical outcome.

**Figure 4:**
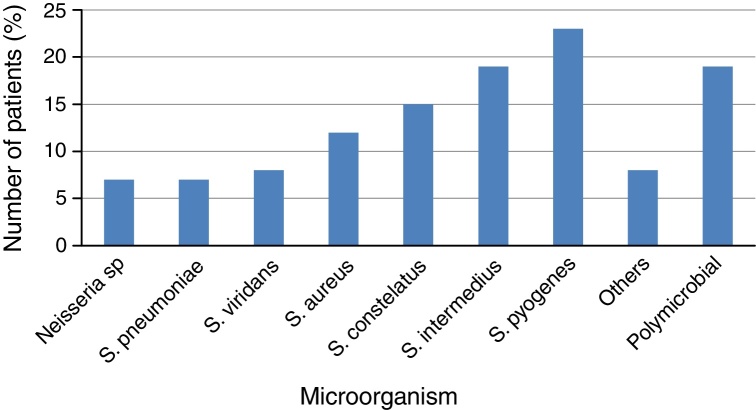
Bacterial growth culture in deep cervical abscesses.

Material for culture was obtained in 76.2% patients. There was no bacterial growth in 14.5% of cases. Polymicrobial culture was detected in 18.8% of patients, being *Streptococcus pyogenes* + *Streptococcus pneumoniae* the most frequent association. *S. pyogenes* was the most common microorganism present in 25 patients (23.3%). The prevalence of other organisms was as follows: *Streptococcus intermedius* (20 patients, 18.6%), *Streptococcus constelattus* (16 patients, 14.9%), *Staphylococcus aureus* (13 patients, 12.1%), *Streptococcus viridians* (9 patients, 8.4%), *Streptococcus pneumonia* (8 patients, 7.4%) and *Neisseria* spp. (7 patients, 6.5%). Other microorganisms (*Corynebacterium* spp., *Eikenella corrodens*, *Enterococcus faecium, Klebsiella pneumoniae* and other *Streptococci*) were less frequent, totaling 12 patients (11.8%). Seventeen patients had clinical signs of sepsis on arrival at the emergency room, and blood tests were made, resulting positive in 13 patients (12.8% of total), with the most prevalent occurrence also of *Streptococcus pyogenes* (Fig. 4).

### Complications, tracheostomy and mortality

The complications of deep neck infection are shown in Table 3. Complications were found in 48 patients (8 children, 40 adults). Of the 8 patients of children with complications, 3 had pneumonia, 3 had Septic shock and 2 had surgical rapprochement. The main complications of cervical abscesses included sepsis (17 patients, 16.8%), pneumonia (11 patients, 10.8%), mediastinitis (2 patients, 1.9%) and jugular vein thrombosis (1 patient, 0.9%). Surgical rapprochement was required in 9 patients (8.9%), probably because of the reorganization of infection in stores (%). The adult patients developed infectious complications more often than the children (*p* = 0.005) (Table 1).

**Table 3:** Complications of deep neck abscesses.

	Children *n* (%) (*n* = 8)^a^	Adults’ *n* (%) (*n* = 40)^a^	All patients (*n* = 101)^a^
Mediastinitis	0	2 (5)	1.98%
Jugular vein thrombosis	0	1 (2.5)	0.99%
Emergency tracheostomy	0	8 (20)	7.92
Pneumonia	3 (37.5)	8 (20)	10.80%
Septic shock	3 (37.5)	14 (35)	16.80%
Surgical rapprochement	2 (25)	7 (17.5)	8.90%

a An individual patient may have 2 or more complications.

The upper airway obstruction and the impossibility of intubation forced the tracheostomy in 17 patients (16.8%). Of these, 8 (7.9%) underwent emergency tracheostomy because of respiratory failure.

There were 2 deaths (1.9% mortality rate). The first case was a 19 year-old healthy female with extensive cervical abscess associated with descending mediastinitis of undetermined etiology. The death occurred on the 3rd postoperative day and she had sepsis with positive blood culture for *Streptococcus pyogenes*. The second case was of a 49 year-old diabetic male with abscess of odontogenic etiology (Ludwig's Angina) extending to the submandibular neck spaces/floor of the mouth and parapharyngeal space. He presented sepsis with polymicrobial blood cultures (*Streptococcus viridians* *+* *Neisseria* spp.).

The average hospital stay was 9.7 days with a variation between 2:45 days. Abscess complications prolonged the hospital stay in about five days (average length of stay of 14.8 days).

## Discussion

Deep neck abscesses are diseases of major importance due to its frequency and severe complications. The incidence is estimated at around 10/100,000 inhabitants/year, with a tendency to increase, especially in children under 5 years,^1^ in whom the estimated incidence is about 2/100,000 inhabitants/year.^1^ The infections have no preference for age or sex and can affect anyone. In agreement with Eftekharian et al. studies, ours observed a higher incidence in the young male population with a mean age of 28.1 years.^8^ However, Huang et al. and other studies are also showing an upward trend in the incidence of infections in older patients and patients with systemic diseases.^9^ In this group, defense mechanisms would be less efficient, recovery rates slower and complications would be more frequent.

Many causes are associated with DCA. In the pre antibiotic era, pharyngeal/tonsillar infections were responsible for 70% of cases of deep neck space infections.^10^ Currently, many studies show a significant decline in that incidence, having odontogenic infections as the most frequent cause.^6^, ^10^, ^11^ In our study, the bacterial tonsillitis was the most common cause (31.68%), followed by odontogenic infection (23.7%), totaling 55.3% of our sample. Other studies have shown an increase associated with intravenous drug abuse and neck trauma infections, although we have not identified these etiologies.^12^

In a study by Coelho et al., dental focus was the origin of abscesses in 37% of patients, whereas pharyngeal tonsil and disorders were present in 20% of cases, not being possible to identify the source of infection in 33% of patients.^13^ To Sennes et al., odontogenic infection was the cause in 42.1% of patients, tonsillitis in 17.5%, post upper airway infections in 15.8% and unknown cause in 8.8%^14^ lymphadenitis. Among other causes, post upper airways infection, lymphadenitis and foreign body ingestion were still found in 17.8% of cases, adenitis (submandibular and parotid) at 7.8% and 39.6% in the fasciitis. Other authors also reported a significant proportion of DCA with unknown primary origin, reaching up to 50% of cases.^2^, ^6^, ^15^, ^16^ In 14.8% of our patients, the infection etiology could not be determined, probably because the initial infection was not promptly diagnosed, although it has preceded the abscess for weeks. Thus, the average time from symptom onset to diagnosis of DCA in our study was 8 days, but reached up to 20 days and the main symptoms were fever and neck pain.

The knowledge of the anatomic relationships between neck spaces is important for the therapeutic management, since the fascia limiting these spaces are important anatomical barriers to the spread of infection, but also serve to direct infection once its natural resistance is overcome.^17^

Most previous studies^1^, ^18^, ^19^ reported that children are a relatively low proportion of their patients with DCA. We found, however, that the proportion of patients who were under 18 years of age were high (27 cases, 26.7%), and none of them had DM or other associated diseases. Other indicators of health care utilization such as medication used before and frequency were not possible to obtain because most patients received treatment for their acute episodes at remote locations, either by their primary care physicians or by the referring otolaryngologist. However, probably this may be caused by the history of antibiotics abuse and the antibiotic resistance explained by indiscriminate use of antibiotics, especially in colds and other viral infections, which are more prevalent in children than in adults.^20^, ^21^ Previous antibiotic use is correlated with higher recovery of resistant organisms and increased incidence of β-lactamase-producing bacteria.^22^, ^23^ Ultimately, the resistance had an effect on the incidence of deep neck infection.^24^ Larger prospective studies are needed to address many of these limitations and to further delineate the role for understanding the different characteristics between the children and adults with deep neck infection, helping in accurate evaluation and proper management.

Most studies^5^, ^25^ reported the retropharyngeal space as the most common involved space in children, but we found the peritonsillar space the most common involved in children group (10–27 patients, 37%), closely followed by parapharyngeal space (9–27 patients, 33.3%). However, there is one study^4^ showed that the parapharyngeal space was as the most common involved space in children. It might be explained beacause infections in the peritonsillar, submandibular, masticatory, and parotid space can usually spread to the parapharyngeal space.^9^ Multispace infection was found in 31 patients (41.8%) in adults and in 2 patients (7.4%) in children (*p* < 0.001). Adults were easier to get multispace infection than children; this might relate to a higher incidence of disease comorbidity in adults. Patients with disease comorbidity tend to have poorer defense against infections and thus result in higher rates of more severe infection in the form of multispace infections.^18^ In both deaths there was involvement of multiple cervical spaces.

The DCA microbiology is characterized by generally being polymicrobial infections, including aerobic and anaerobic, gram positive particularly. Among the agents commonly found are: *Streptococcus viridans*, *Streptococcus milleri*, *Prevotella* spp., *Peptosstreptococcus* spp*.* and *Klebisiella pneumoniae*, the latter being more common in diabetic patients.^26^ Sennes found *Streptococcus viridans* in 41.5% of the cases, *Staphylococcus aureus* in 20.7% of them, and 3.8% with *Haemophilus influenza*.^14^ In our study, *Streptococcus pyogenes* was the most frequently detected agent (23.3%), which can be explained by the higher incidence of peritonsillar infections. From all patients, 14.5% showed no growth of bacteria in culture, which can be explained by the use of high doses of intravenous antibiotics before abscess surgical drainage.^27^ In 12.8% of patients, it was detected positive blood culture, which shows the high possibility of systemic spread of an infection initially uncontrolled.

Many studies have shown the association of DM with DCA.^9^, ^28^ Huang et al.^9^, ^20^ and Lee et al.^18^ indicated that old patients with DM were susceptible to deep neck infection. In DM patients, hyperglycemia may impair several mechanisms of humoral host defense, such as varied neutrophil functions: adhesion, chemotaxis and phagocytosis and result in predisposition to infection and complications.^29^ Huang et al. found high rates of infection with *Klebsiella pneumoniae* in diabetic patients.^9^ The prevalence of diabetic patients in our study was low (12.8%), with *Staphylococcus aureus* (5 patients) and *Streptococcus pyogenes* (4 patients) as the microorganisms most commonly found. In one of the lethal developments, the patient with poorly controlled diabetes had the blood culture positive for *Streptococcus viridians* *+* *Neisseria* spp.

The management of DCA involves surgical drainage associated with use of intravenous antibiotics. Computed tomography with contrast was the test of choice for diagnosing and assessing the abscess extent. Although it has high sensitivity, the test specificity is low, for example in cases of lymph node clusters without associated abscess, which can lead to unnecessary surgical procedures.^30^ Oh et al. and other researchers have demonstrated the efficacy of needle drainage of abscess guided by ultrasound, with no increase in complication rates.^27^ In general, this conservative treatment is effective in small collections and with no evidence of imminent complications. Our service has little experience in this treatment, preferring the open surgical drainage in all cases.

Antibiotic therapy was empirically initiated before culture and sensitivity results being available. The choice of amoxicillin plus clavulanic acid as first-line treatment in 82.1% of cases was based on the coverage of bacteria commonly found in our environment, both positive and anaerobic gram. Many authors advise, for an optimal empirical coverage, a penicillin with a β-lactamase inhibitor combination (such as amoxicillin and clavulanic acid), or a β-lactam antibiotic-resistant (such as cefuroxime, meropenem or imipenem) in combination with a drug, which is highly effective against most anaerobes (such as metronidazole or clindamycin).^17^, ^28^ Afebrile after 48 h, the patient was discharged with a prescription of oral antibiotic.

Despite the widespread use of antibiotics, several undesirable and life-threatening complications of DCA are known as descending mediastinitis, jugular vein thrombosis, pericarditis, pleural empyema, arterial erosion, upper airway obstruction and sepsis. In our study, there were two cases of mediastinitis, both in adults and involving multiple neck spaces, and death in septic shock in one of them (Table 1). In mediastinitis, the patient often complains of increased chest pain or dyspnea. The extent of disease occurs via anterior visceral space and mortality can reach half of the cases, requiring combined thoracic drainage.^31^ The obstruction of the upper airway and consequent respiratory failure forced the emergency tracheostomy in 7.9% of patients. Har-El et al. describe that the involvement of the floor of the mouth and the retropharyngeal space are more associated with airway obstruction and greater need for tracheostomy, procedure necessary in 75% of the cases.^10^ It is interesting to note that in our study, especially in patients with secondary involvement of the masticatory muscles, trismus, tracheostomy was necessary because of the impossibility of intubation, even without the presence of respiratory failure. Our mortality rate was 1.9%, similar to that described by Huang et al. (1.6%).^9^

We presented here relevant information about clinical and surgical DCA outcomes. However, the course and severity of the same infection in different patients can vary widely, requiring an experienced team to deal with it.

## Conclusions

Deep neck infections constitute a medical and surgical emergency. The clinical features and severity of DCA varied according to different age groups, perhaps due to the location of the infection and a higher incidence of comorbidity in adults. Thus, DCA in adults is more facile to have multispace involvement and lead to complications and seems to be more serious than that in children.

## Conflicts of interest

The authors declare no conflicts of interest.
